# Cation Valences and Multiferroic Properties of EuTiO_3_ Co-Doped with Ba and Transition Metals of Co/Ni

**DOI:** 10.3390/ma15196652

**Published:** 2022-09-25

**Authors:** Tzu-Chiao Lin, Xiaoding Qi

**Affiliations:** 1 Department of Materials Science and Engineering, National Cheng Kung University, Tainan City 70101, Taiwan; 2 Center for Micro/Nano Science and Technology, National Cheng Kung University, Tainan City 70101, Taiwan

**Keywords:** europium titanate, EuTiO_3_, ferroelectric, ferromagnetic, multiferroic

## Abstract

Eu_1−*x*_Ba*_x_*Ti_1−*y*_M*_y_*O_3_ (M = Co or Ni) was sintered at 1400 °C under a reduction atmosphere. X-ray photoelectron spectroscopy revealed the mixed valences of Eu^2+^/Eu^3+^ and Ti^4+^/Ti^3+^ in EuTiO_3_ and Eu_0.7_Ba_0.3_TiO_3_, as well as some oxygen vacancies required to keep the charge neutrality. The co-doping of Co^2+^/Ni^2+^ in Eu_0.7_Ba_0.3_TiO_3_ resulted in the disappearance of oxygen vacancies, as a result of a reduction in Ti^3+^ numbers and an increase in Eu^3+^ numbers. On the other hand, Ba^2+^ doping led to an increased lattice parameter due to its larger ionic size than Eu^2+^, whereas the Co^2+^/Ni^2+^ co-doping resulted in smaller lattice parameters because of the combined effects of ionic size and variation in the oxygen-vacancy numbers. Eu_0.7_Ba_0.3_TiO_3_ exhibited a clear ferroelectricity, which persisted in the Co^2+^/Ni^2+^ co-doped samples until the doping levels of *y* = 0.05 and 0.10, respectively. Eu_0.7_Ba_0.3_TiO_3_ remained to be antiferromagnetic with a reduced transition temperature of 3.1 K, but co-doping of Co^2+^/Ni^2+^ turned the samples from antiferromagnetic to ferromagnetic with transition temperatures of 2.98 K and 2.72 K, respectively. The cause for such a transition could not be explained by the larger lattice volume, oxygen vacancies and mixed valences of Eu^2+^/Eu^3+^, which were proposed in previous works. Instead, it was more likely to arise from a large asymmetric distortion of the Eu–O polyhedron introduced by the aliovalent doping, which promotes the admixture of Eu 5d and 4f states.

## 1. Introduction

Pristine and doped EuTiO_3_, in both bulk and thin-film forms, have been studied intensively during the past ten years or so due to a variety of interesting properties including multiferroicity [1,2,3], hidden spin orders [4,5,6], large magnetodielectric and magnetocaloric effects [7,8,9,10], etc. EuTiO_3_ belongs to the perovskite-structured oxides, which has an undistorted cubic lattice at room temperature (RT). Pristine EuTiO_3_ is known as an incipient ferroelectric with a large dielectric constant (~400) at low temperature [7], and a G-type antiferromagnet below 5.3 K [11]. It was predicted by first-principles calculation to exhibit strong ferromagnetism and ferroelectricity simultaneously under large biaxial compressive strains [12]. However, a later theoretical work indicated that the multiferroicity may also be induced by biaxial tensions with the added benefit of less strain required, which was demonstrated experimentally in epitaxial EuTiO_3_ thin films [3]. Apart from the heteroepitaxial thin films, structural distortions and strains may also be introduced by chemical doping. EuTiO_3_ has therefore been substituted at both the A-site (Eu^2+^) and B-site (Ti^4+^) for a variety of interests. The dopants at A-site included alkaline-earths, such as Sr, Ba and Ca [10,13,14,15,16,17], as well as rare-earths other than Eu (e.g., La, Ce and Sm) [17,18], whereas the dopants at B-site were mainly transition metals (TMs) (e.g., Cr, Ni, Mn, Fe, Co, Nb, Zr, etc.) [19,20,21,22,23,24] and a few main group-III elements (e.g., Al and Ga) [25]. 

Apart from Ba, doping of other elements at either the A or B site has so far been unable to turn EuTiO_3_ into a real ferroelectric. The appearance of ferroelectricity in the Ba-doped EuTiO_3_ is not surprising. BaTiO_3_ (a typical ferroelectric) is isostructural to EuTiO_3_, allowing it to form a full range of solid solutions with EuTiO_3_. So, at a high enough *x*, Eu_1−*x*_Ba*_x_*TiO_3_ must become ferroelectric [13,14]. In contrast to the “inertness” of ferroelectricity to chemical doping, the magnetic properties of EuTiO_3_ are affected notably by the chemical doping at either the A or B site. In particular, doping of either TMs or main group elements at the B-site by ions of lower valence than Ti^4+^, such as Cr^3+^, Ni^2+^, Mn^2+^, Fe^3+^, Co^2+^, Al^3+^ and Ga^3+^, results in ferromagnetism [19,20,21,22,23,25], whereas isovalent doping at the B site by tetravalent ions, such as Zr^4+^, only leads to a decrease in antiferromagnetic ordering temperature (T_N_) over the entire substitution range of EuTi_1−*x*_Zr*_x_*O_3_, i.e., T_N_ = 5.6 K at *x* = 0 to T_N_ = 4.1 K at *x* = 1 [24]. Furthermore, the effect of doping higher-valence ions at the B site was found to be dependent on the dopant concentration. It was showed that doping of ≤5 at% Nb^5+^ resulted in a similar decrease in T_N_ as in the case of isovalent doping [24]. However, higher doping level (>5 at%) of Nb^5+^ led to ferromagnetism, and this was accompanied by a transition from the semiconductor conductivity to metallic conductivity [24], tempting the speculation that the ferromagnetism may arise from the itinerant d-electron-mediated Eu 4f spin interactions. Such a mechanism for ferromagnetism in the donor-doped EuTiO_3_ has also been proposed for the La^3+^-doped EuTiO_3_ at the A-site, which introduced electrons to the empty Ti 3d states [18]. On the other hand, as with the isovalent doping at the B site, doping at the A site by high concentrations of isovalent ions, such as Ba^2+^ and Ca^2+^, does not switch EuTiO_3_ from an antiferromagnet to a ferromagnet; i.e., Eu_1−*x*_Ba*_x_*TiO_3_ remains antiferromagnetic with a reduced T_N_ of 1.9 K at *x* = 0.5 [14], while Eu_1−*x*_Ca*_x_*TiO_3_ is also antiferromagnetic at *x* = 0.6 with T_N_ of 2.6 K [15].

Although there have been a number of studies on the chemical doping at A or B sites of EuTiO_3_, the studies on the co-doping at both A and B sites are far fewer in comparison. In this work, we investigated the effects of Ba and TM co-doping on the ferroelectric and magnetic properties of EuTiO_3_, as well as the changes in cation valences in the co-doped EuTiO_3_. The stable valence of Eu in air is 3+ whereas the Eu valence in EuTiO_3_ is 2+. So, EuTiO_3_ has to be synthesized in a sufficient reduction atmosphere to avoid Eu^3+^, which may also reduce some Ti^4+^ in EuTiO_3_ to lower valence Ti^3+^. Therefore, apart from Eu^2+^ and Ti^4+^, a certain amount of Eu^3+^ and/or Ti^3+^ are expected to coexist in the samples, but they have not been well studied experimentally so far. It is well-known that the magnetic property in EuTiO_3_ mainly arises from Eu^2+^, which has seven unpaired 4f electrons. So, in order to retain strong magnetic interaction, doping at the Eu^2+^ site was carried out in this work with the minimum amount of Ba^2+^ that was required to induce a clear ferroelectricity, and then the TM co-doping at the Ti^4+^ site was taken to the highest possible concentration, above which a secondary phase might occur or the ferroelectricity might disappear. The ferroelectric and magnetic properties, which coexisted in such co-doped EuTiO_3_ of a pure phase, were then investigated in detail. 

## 2. Materials and Methods

Eu_1−*x*_Ba*_x_*Ti_1−*y*_M*_y_*O_3_ (M = Co or Ni, *x* = 0–0.3 and *y* = 0–0.1) samples were synthesized by solid-state reaction in an air-tight tube furnace filled with a mixed-gas stream of Ar + 3%H_2_, flowing at 50 sccm. The starting chemicals included the powders of Eu_2_O_3_ (99.9%, Alfa Aesar), TiO_2_ (99.9%, Showa), BaCO_3_ (99.95%, Alfa Aesar), Co_3_O_4_ (99.7%, Alfa Aesar) and NiO (99.8%, Showa). The powders were weighed according to the stoichiometric ratios of the metallic elements, and well-mixed by wet ball-milling with the addition of ethanol. The mixture was then dried and pressed into pellets measuring about 8 mm in diameter by 1–2 mm thick, under a pressure of 50 Kg/cm^2^ with the addition of 1.0 wt% PVB (polyvinyl butyral) as the binder, which was in the form of 10 wt% ethanol solution. The pellets were heated to 650 °C at 5 °C/min and dwelled for 1 h to remove the PVB binder. Afterwards, they were sintered at 1400 °C for 20 h. The synthesized samples were characterized by a range of techniques, including powder X-ray diffraction (XRD, Bruker D2 Phaser), X-ray photoelectron spectroscopy (XPS, PHI 5000 VersaProbe), and the measurements of DC conductivity (Agilent 34410A), ferroelectric hysteresis (Radiant Precision LC Ferroelectric Tester) and magnetic properties (Quantum Design MPMS). In the ferroelectric and DC conductivity measurements, both surfaces of the circular pellets were painted with silver paste as the electrodes.

## 3. Results and Discussion

### 3.1. Phase Purity, Structure and Valences of Cations

**Figure 1:**
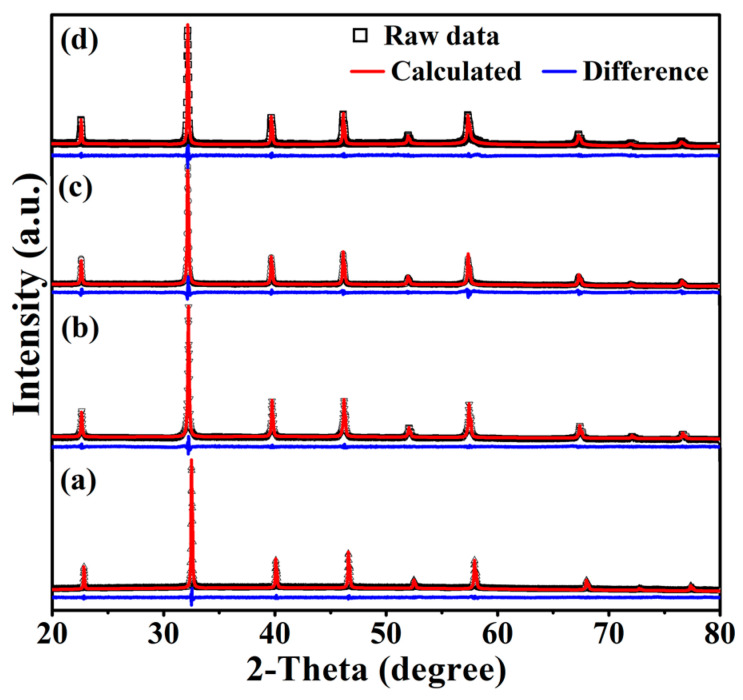
XRD patterns and TOPAS fittings: (**a**) EuTiO_3_, (**b**) Eu_0.7_Ba_0.3_TiO_3_, (**c**) Eu_0.7_Ba_0.3_Ti_0.90_Co_0.10_O_3_ and (**d**) Eu_0.7_Ba_0.3_Ti_0.90_Ni_0.10_O_3_.

Figure 1 shows the XRD patterns of Eu_1−*x*_Ba*_x_*Ti_1−*y*_M*_y_*O_3_ recorded at RT. All the reflection lines of each sample can be identified with the known EuTiO_3_ phase in the powder diffraction database (PDF#73-2120), which is cubic with the space group Pm-3m. The results indicate that all the synthesized samples had a pure phase and the co-substitutions of 30 at% Eu by Ba and 10 at% Ti by Co/Ni did not incur segregation of any secondary phase. However, the substitutions did result in structural variations, as indicated by the shifts of reflection lines. Thus, the actual crystal structures of the synthesized samples were refined based on the XRD data by the TOPAS software [26], which combines a number of fitting techniques including the Rietveld refinement. Table 1 lists the refined lattice parameters of the samples. The undoped EuTiO_3_ had a lattice parameter of 3.8976 Å, almost the same as that given in PDF#73-2120. Doping of 30 at% Ba^2+^ (i.e., Eu_0.7_Ba_0.3_TiO_3_) resulted in a larger lattice parameter of 3.9340 Å, which is expected in view of the larger ionic size of Ba^2+^ (1.52 Å) compared to Eu^2+^ (1.35 Å). Similar increases in the lattice parameters of Eu_1−*x*_Ba*_x_*TiO_3_ were also reported elsewhere [13]. 

However, the variations in lattice parameter induced by Co/Ni co-doping at the Ti^4+^ site were more complicated due to aliovalent substitutions. Apart from the ionic size, structural changes incurred by charge-compensation mechanisms might have to be taken into account. As will be discussed below, although the valence states of Eu and Ti in EuTiO_3_ are usually assumed to be +2 and +4, respectively, XPS analyses indicated the existence of Eu^3+^ and Ti^3+^, whose amounts varied with the co-doping of Co or Ni, which were both in the valence states of +2. As indicated in Table 1, both Co^2+^ and Ni^2+^ co-doping at Ti^4+^ sites resulted in smaller lattice parameters compared to Eu_0.7_Ba*_0.3_*TiO_3_, as the consequence of a reduced number of Ti^3+^ and the correlated oxygen vacancies. The latter is known to cause a larger size of lattice, i.e., the so-called chemical expansion [27,28], so a reduction of such will result in a smaller lattice size.

**Figure 2:**
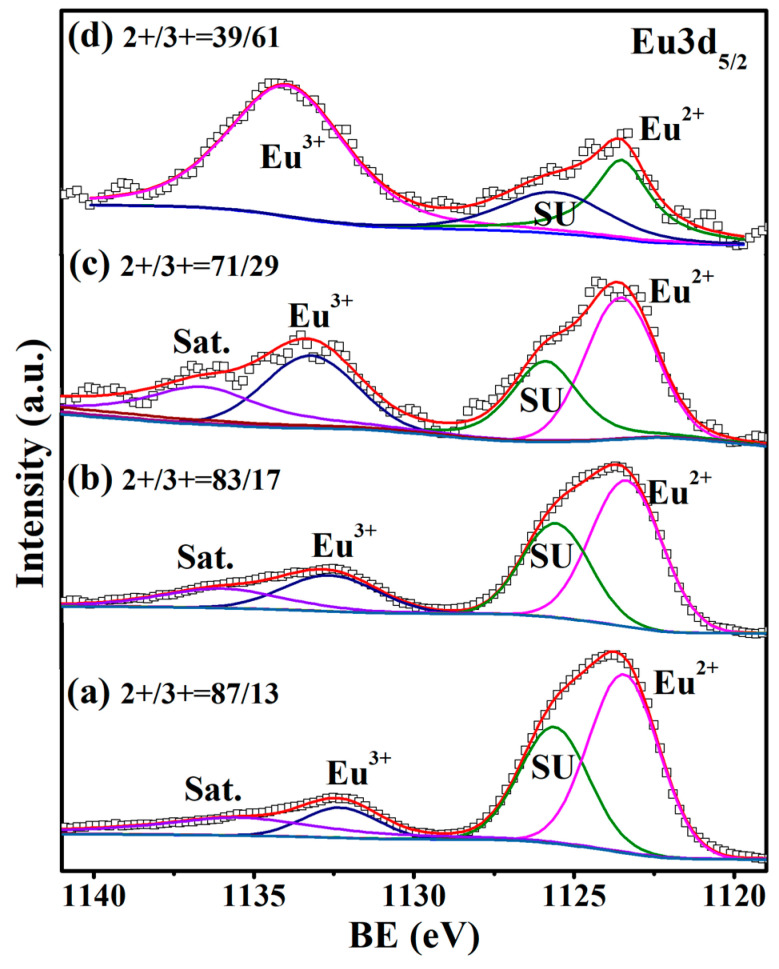
Eu 3d_5/2_ XPS spectra: (**a**) EuTiO_3_, (**b**) Eu_0.7_Ba_0.3_TiO_3_, (**c**) Eu_0.7_Ba_0.3_Ti_0.95_Co_0.05_O_3_ and (**d**) Eu_0.7_Ba_0.3_Ti_0.90_Ni_0.10_O_3_. SU: shake-up peak (often seen with paramagnetic ions including Eu^2+^ [32]).

**Figure 3:**
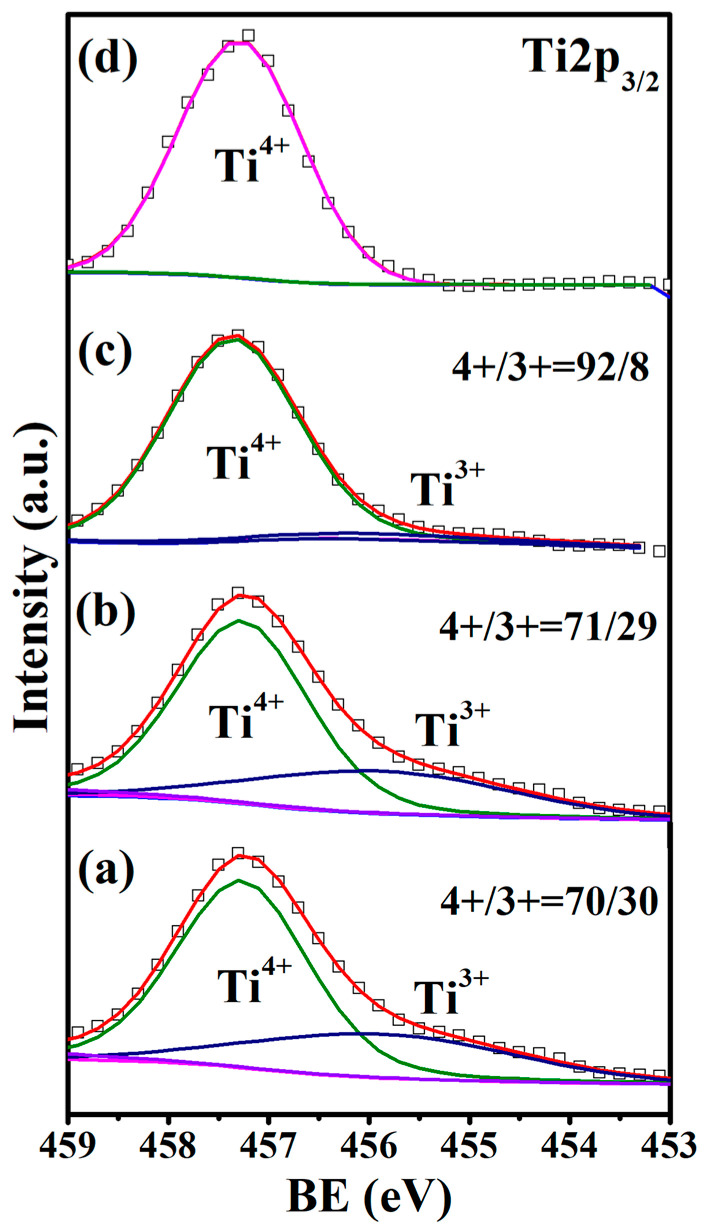
Ti 2p_3/2_ XPS spectra: (**a**) EuTiO_3_, (**b**) Eu_0.7_Ba_0.3_TiO_3_, (**c**) Eu_0.7_Ba_0.3_Ti_0.95_Co_0.05_O_3_ and (**d**) Eu_0.7_Ba_0.3_Ti_0.90_Ni_0.10_O_3_.

Figure 2 shows XPS spectra around the Eu 3d_5/2_ binding energy (BE) for Eu_1−*x*_Ba*_x_*Ti_1−*y*_M*_y_*O_3_. All the samples contained some amounts of Eu^3+^, despite the fact that they were synthesized under the reduction atmosphere described in the experimental section. The rare-earth elements are usually trivalent, so the existence of some Eu^3+^ in EuTiO_3_ might be expected and was, indeed, also observed in previous works [29,30]. The charge imbalance due to the occurrence of Eu^3+^ was compensated for by the presence of Ti^3+^ in the samples, as indicated in the Ti 2p_3/2_ XPS spectra (Figure 3). The ratios of Eu^2+^/Eu^3+^ and Ti^4+^/Ti^3+^ can be estimated by the ratios of peak area for each ion, which were 87/13 and 70/30, respectively, for undoped EuTiO_3_, indicating that the number of Ti^3+^ was more than that needed for charge compensation of Eu^3+^. So, a small amount of oxygen vacancies was required to keep an overall charge neutrality, which was calculated to be 2.97% (i.e., EuTiO_2.91_). Ba is known to have a stable valence state of +2, which was confirmed in XPS. The isovalent substitution of Ba^2+^ with Eu^2+^ caused little change in the Eu^2+^/Eu^3+^ and Ti^4+^/Ti^3+^ ratios, as seen in Figure 2b and Figure 3b. So, the Ba^2+^-doped samples had approximately the same oxygen deficiency (2.95%) as undoped EuTiO_3_. Such an amount of oxygen vacancies was in the range reported for the epitaxial films of Eu_0.5_Ba_0.5_TiO_3−δ_ (δ = 0.02–0.15) grown under low oxygen partial pressures (10^−1^–10^−4^ Pa) [31].

**Figure 4:**
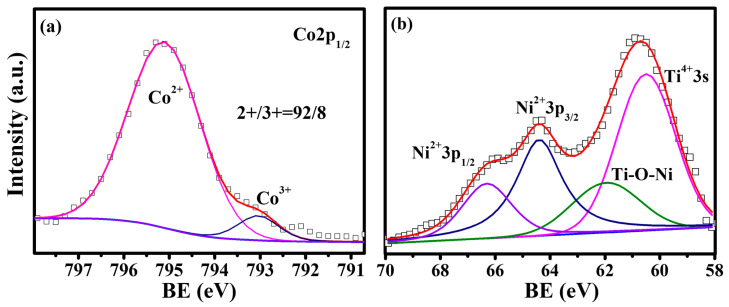
(**a**) Co 2p_1/2_ XPS spectrum and (**b**) Ni 3p XPS spectrum (note: Ti 3s peaks also located in this BE range, see discussion in text).

Figure 4a shows the XPS spectrum around the BE of Co 2p_1/2_ for Eu_0.7_Ba_0.3_Ti_0.95_Co_0.05_O_3_, which consists of a main peak at 795.1 eV and a small shoulder at the lower BE side (793.0 eV). Co is one of the known elements whose valence and chemical shift in XPS are reversed, so the main peak arose from Co^2+^, while the small shoulder was the contribution from Co^3+^ [33,34]; i.e., the dominant valence of Co in the co-doped samples was +2 (Co^2+^/Co^3+^ ≈ 92/8). Figure 4b shows the XPS spectrum around the BE of Ni 3p for Eu_0.7_Ba_0.3_Ti_0.90_Ni_0.10_O_3_, in which the two peaks at 66.31 and 64.40 eV with a separation of 1.9 eV were identified with the Ni^2+^ 3p_1/2_ and 3p_3/2_ doublet, according to the previously reported BEs for divalent Ni oxides such as NiO and La_2_NiMnO_6_ [35,36,37]. It is noted that the BE of Ti 3s also falls in the range of Figure 4b and the peak at 60.5 eV matches well with the reported values for Ti^4+^ 3s [38,39]. This peak has to be fitted with a small peak at 61.96 eV, which may come from those Ti^4+^ ions with the dopant Ni^2+^ at neighboring sites. The 3s sublevel is in the outermost atomic shell of Ti^4+^, so its BE is susceptible to the change in surrounding ions.

In contrast to the isovalent doping of Ba^2+^ at the Eu^2+^ site, the aliovalent doping of Co^2+^/Ni^2+^ at the Ti^4+^ site resulted in notable changes in both the Eu^2+^/Eu^3+^ and Ti^4+^/Ti^3+^ ratios, as compared in Figure 2 and Figure 3. The Eu^2+^/Eu^3+^ ratio decreased whereas the Ti^4+^/Ti^3+^ ratio increased, i.e., more Eu^3+^ and less Ti^3+^ were generated in the Co^2+^ or Ni^2+^ co-doped samples. Particularly in Eu_0.7_Ba_0.3_Ti_0.90_Ni_0.10_O_3_, the amount of Eu^3+^ was more than Eu^2+^, and yet, the BE of Ti 2p_3/2_ was fit very well by a single peak for Ti^4+^, indicating the absence of Ti^3+^. The increase in Eu^3+^ numbers and decrease in Ti^3+^ numbers in the Co^2+^/Ni^2+^ co-doped samples alluded to the need for small amount of oxygen interstitials instead of oxygen vacancy for charge compensation, i.e., Eu_0.7_Ba_0.3_Ti_0.95_Co_0.05_O_3.10_ and Eu_0.7_Ba_0.3_Ti_0.90_Ni_0.10_O_3.13_. However, it is noted that other charge-compensation mechanisms than the oxygen interstitials for a higher overall cation valence in the formula may also be possible (e.g., slight variation in the A/B-site cation ratio).

### 3.2. Ferroelectric Properties

**Figure 5:**
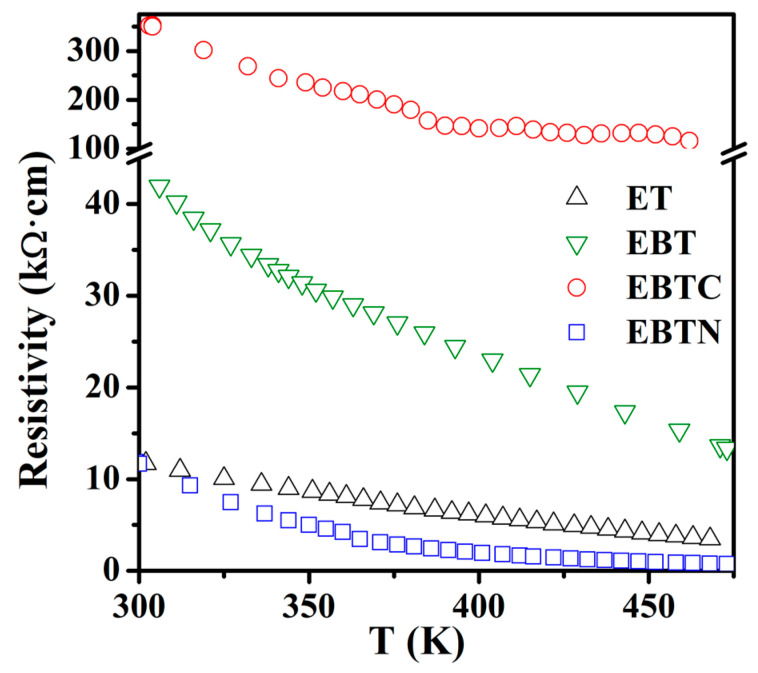
DC resistivity vs. temperature, ET: EuTiO_3_, EBT: Eu_0.7_Ba_0.3_TiO_3_, EBTC: Eu_0.7_Ba_0.3_Ti_0.95_Co_0.05_O_3_, EBTN: Eu_0.7_Ba_0.3_Ti_0.90_Ni_0.10_O_3_.

**Figure 6:**
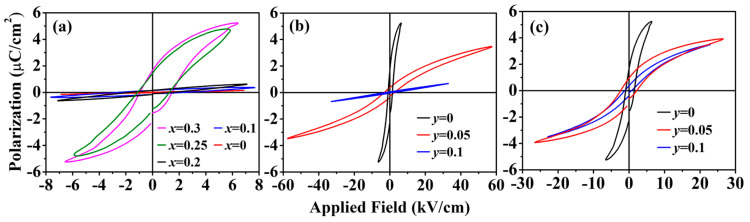
P–E hysteresis loops measured at 77 K with frequency of 1 kHz: (**a**) Eu_1__−*x*_Ba*_x_*TiO_3_ (*x* = 0–0.3), (**b**) Eu_0.7_Ba_0.3_Ti_1__−*y*_Co*_y_*O_3_ (*y* = 0, 0.5 and 0.1) and (**c**) Eu_0.7_Ba_0.3_Ti_1__−*y*_Ni*_y_*O_3_ (*y* = 0, 0.5 and 0.1).

The Eu_1−*x*_Ba*_x_*Ti_1−*y*_M*_y_*O_3_ samples synthesized in this work had a high resistivity (>10 kΩ·cm at RT), which decreased with an increase in temperature (i.e., a nonmetallic behavior), as shown in Figure 5. Nevertheless, the polarization vs. field (P–E) measurements at RT were still complicated by the leakage current driven under the large applied field that was required to switch ferroelectric domains. However, as discussed in Section 3.1, all the samples had a cubic structure at RT; therefore, they should not be ferroelectric at RT. So, the measurements were taken at 77 K. Figure 6a shows the P–E hysteresis loops for Eu_1−*x*_Ba*_x_*TiO_3_ (*x* = 0–0.3). The samples with *x* < 0.25 exhibited linear P–E curves, indicating a paraelectric behavior. Typical P–E hysteresis loops for the ferroelectric behavior occurred with the samples of *x* ≥ 0.25, which was consistent with the previous report [13]. However, it is noted that the ferroelectricity in the *x* = 0.25 samples was observed at a temperature below 60 K in the previous work [13]. Such a difference in transition temperature (T_C_) might arise from the small deviation in actual sample compositions, because T_C_ of Eu_1−*x*_Ba*_x_*TiO_3_ (*x* ≥ 0.25) was found to shift greatly with *x*, i.e., T_C_ ≈ 60 K at *x* = 0.25, which shifted to T_C_ ≈ 145 K at *x* = 0.35, and to T_C_ ≈ 196 K at *x* = 0.45 [13].

Because *x* = 0.25 was the lowest Ba-doping level to induce ferroelectricity in Eu_1−*x*_Ba*_x_*TiO_3_ at 77 K, a slightly higher Ba-doping level, i.e., *x* = 0.30, was then chosen to study the Co/Ni co-doping effect on the ferroelectricity of Eu_0.7_Ba_0.3_Ti_1−*y*_M*_y_*O_3_ (M = Co or Ni). Figure 6b shows the P–E hysteresis loops of Eu_0.7_Ba_0.3_Ti_1−*y*_Co*_y_*O_3_, which indicate that the ferroelectricity remained in the samples of *y* = 0.05, whereas in the *y* = 0.10 samples the ferroelectricity had disappeared. On the other hand, Eu_0.7_Ba_0.3_TiO_3_ allowed higher Ni co-doping at Ti-site and a ferroelectricity could still be observed in the Eu_0.7_Ba_0.3_Ti_1−*y*_Ni*_y_*O_3_ samples of *y* = 0.10. However, the co-doping of either Co or Ni led to a reduced remanent polarization (P_r_), as shown in Table 2, indicating the weakening of ferroelectricity. The result implies that, similar to BaTiO_3_, the ferroelectricity in Eu_0.7_Ba_0.3_TiO_3_ may arise from the so-called d^0^ mechanism, in which the spontaneous polarization is created by the hybridization between the filled oxygen 2p states and the empty d states of the TM cation [40]. Substitution of non-d^0^ ions, such as Co^2+^ and Ni^2+^, reduces net charge transfer between TM d and O p states and thus leads to the weaker ferroelectricity. However, it is not clear why ferroelectricity existed in higher Ni^2+^ co-doped samples than in the Co^2+^ co-doped samples. Although this may be related to different crystal field splitting and electronic occupation of d^7^ (Co^2+^) and d^8^ (Ni^2+^) states, theoretical calculation is needed for a better understanding, which is apparently outside the scope of the current work.

### 3.3. Magnetic Properties

**Figure 7:**
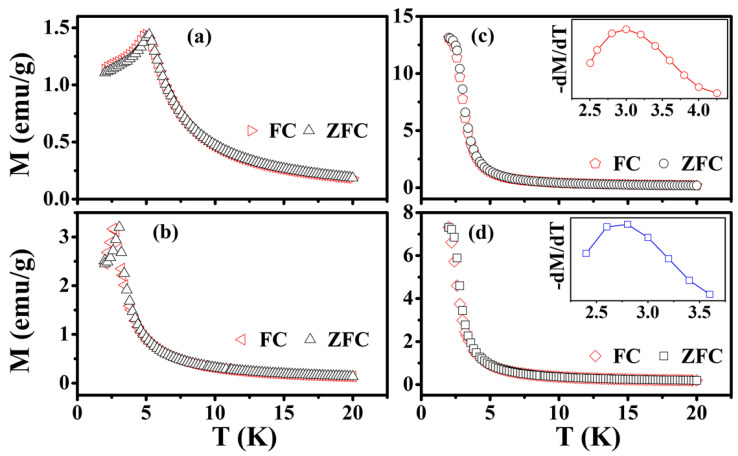
M(T) measured under H = 100 Oe after ZFC and FC: (**a**) EuTiO_3_, (**b**) Eu_0.7_Ba_0.3_TiO_3_, (**c**) Eu_0.7_Ba_0.3_Ti_0.95_Co_0.05_O_3_ and (**d**) Eu_0.7_Ba_0.3_Ti_0.90_Ni_0.10_O_3_. Inset in (**c**,**d**) is minus the first derivative of M(T).

Figure 7 shows temperature-dependent magnetization, M(T), of Eu_1−*x*_Ba*_x_*Ti_1−*y*_M*_y_*O_3_ measured under a small applied field of 100 Oe. There was little difference between the M(T) curves measured after zero field cooling (ZFC) and field cooling (FC) for all the samples, indicating no complication from field-induced effects. The M(T) curve of EuTiO_3_ was characterized by a cusp at 5.2 K, which is typical of an antiferromagnet. Eu_0.7_Ba_0.3_TiO_3_ displayed a similar M(T), but the cusp occurred at a lower temperature (i.e., 3.1 K). In contrast, the M(T) curves of Co/Ni co-doped samples showed a different character, i.e., M(T) started to increase rapidly at the temperature of ~4 K and then saturated at T < 2.5 K (there is an inflection point between 2–4 K), which is the behavior of a ferromagnet. The results suggest that although the substitution of Eu^2+^ by Ba^2+^ only resulted in a reduction in T_N_, agreeing with the previous reports [14,41], the Co^2+^/Ni^2+^ co-doping at Ti^4+^-site turned the co-doped samples into ferromagnets, with the Curie temperatures (T_C_) being 2.98 K and 2.72 K, respectively, which were taken at the peak of first derivative of M(T), i.e., the inflection point, as shown in the insets of Figure 7c,d.

**Figure 8:**
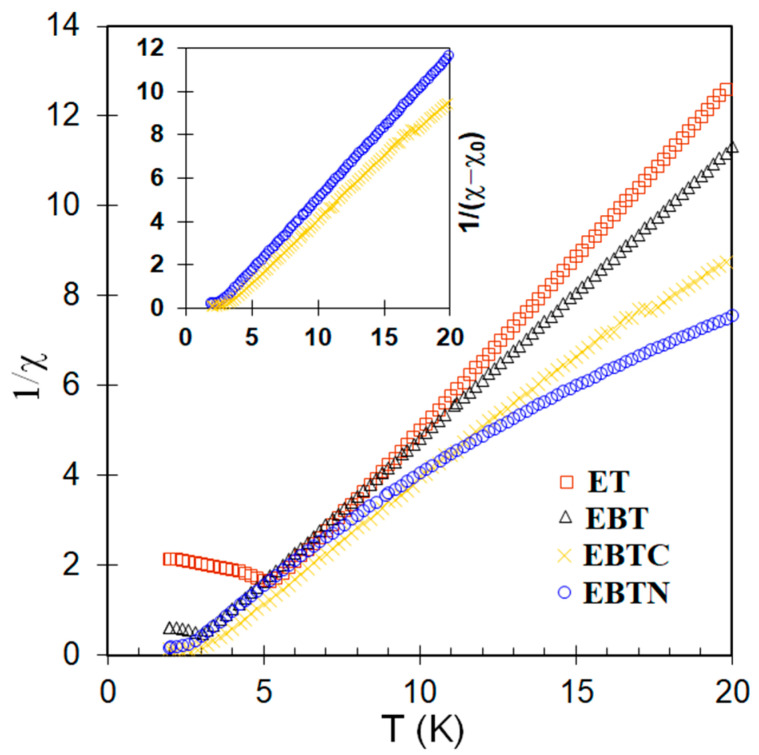
1/χ vs. T graphs. ET: EuTiO_3_, EBT: Eu_0.7_Ba_0.3_TiO_3_, EBTC: Eu_0.7_Ba_0.3_Ti_0.95_Co_0.05_O_3_, EBTN: Eu_0.7_Ba_0.3_Ti_0.90_Ni_0.10_O_3_. Inset: 1/(χ–χ_0_) vs. T for EBTC and EBTN.

Figure 8 shows the graphs of inverse magnetic susceptibility vs. temperature (χ^−1^–T), which are linear for EuTiO_3_ and Eu_0.7_Ba_0.3_TiO_3_ at T > T_N_, indicating that the Curie–Weiss law is followed. In contrast, there is a clear deviation from the linearity in χ^−1^–T at T > T_C_ for Co^2+^/Ni^2+^ co-doped samples. However, as shown in the inset of Figure 8, it was found that the linearity could be well-restored by adding a temperature-independent term (χ_0_) to the Curie–Weiss law, i.e., χ = χ_0_ + C/(T − θ), where C is the Curie constant and θ is the paramagnetic Curie temperature. The fitted values of χ_0_ and θ, together with the T_C_ calculated from –dM(T)/dT, are listed in Table 3. The origin of χ_0_ might arise from the trivalent Eu^3+^, which was known to have a large Van Vleck temperature-independent paramagnetism at low temperature (<100 K), such as in the cases of EuBO_3_, EuF_3_ and Eu_2_O_3_ [42]. Indeed, as shown in Figure 2, the Co^2+^/Ni^2+^ co-doping led to a great increase in the numbers of Eu^3+^. In particular, in the 10 at% Ni^2+^ co-doped samples, the number of Eu^3+^ was more than Eu^2+^, so these samples had a large χ_0_ (see Table 3) and their χ^−1^–T curve in Figure 8 deviated the most from the linearity. Although Figure 2 shows that Eu^3+^ was also present in EuTiO_3_ and Eu_0.7_Ba_0.3_TiO_3_, the numbers were probably too small to have a notable contribution.

**Figure 9:**
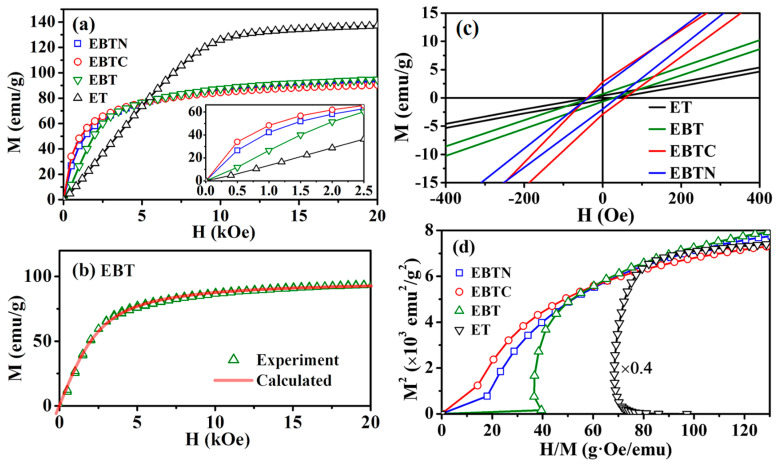
(**a**) M−H measured at 2 K. ET: EuTiO_3_, EBT: Eu_0.7_Ba_0.3_TiO_3_, EBTC: Eu_0.7_Ba_0.3_Ti_0.95_Co_0.05_O_3_, EBTN: Eu_0.7_Ba_0.3_Ti_0.90_Ni_0.10_O_3_. Inset: magnified part at H ≤ 2.5 kOe. (**b**) Brillouin function fitting for EBT’s M−H: red line is the fitting, and green triangles are the experiment points. (**c**) Magnified part of M−H loops around the origin, showing the larger remanences of EBTC and EBTN. (**d**) Arrott plots (M^2^ vs. H/M), note: the graph for ET is multiplied by a factor of 0.4 in order to plot in a same scale.

Figure 9a shows the magnetization vs. field (M–H) curves measured at 2 K, in which the M of EuTiO_3_ has a linear H dependence until H ≈ 7.5 kOe and then saturates at higher H. This confirms that EuTiO_3_ is, indeed, an antiferromagnet. When H is applied to an antiferromagnet, M increases linearly with H as the spin arrangement changes from antiferromagnetic to the spin-flop state, and then the angle between H and the flopped spins decreases until zero at a sufficiently high H, leading to a field-induced ferromagnetic state. Figure 9a shows that Eu_0.7_Ba_0.3_TiO_3_ also has a portion of linear M–H, which ends at a much lower H (<2 kOe), indicating a much weaker exchange interaction among the Eu ions as a consequence of the dilution by Ba-doping. This is supported by the lower T_N_ observed in Figure 7 for Eu_0.7_Ba_0.3_TiO_3_. Furthermore, it was found that the whole M–H curve of Eu_0.7_Ba_0.3_TiO_3_ could be fitted by the Brillouin function (see Figure 9b), confirming the weakness of the exchange interaction, because such a magnetization process is for paramagnets in which the exchange interaction is negligible. The M–H curves of either EuTiO_3_ or the Co/Ni co-doped samples could not be fitted by the Brillouin function due to stronger exchange interactions. 

The Co/Ni co-doped samples do not display any linear portion in the M–H graph, as highlighted in the inset of Figure 9a, which also shows that their M–H curves lie above the linear part of the curve for Eu_0.7_Ba_0.3_TiO_3_, i.e., they have larger M under small H (<2500 Oe) due to the enhancement from ferromagnetic interaction. The M–H curves were measured at 2 K, which was only slightly lower than T_C_ of the co-doped samples. So, their remanences were small, but nevertheless, the remanences of the Co/Ni co-doped samples were still much larger compared to EuTiO_3_ and Eu_0.7_Ba_0.3_TiO_3_, as shown in Figure 9c. To further confirm that the Co/Ni co-doping had turned the samples from antiferromagnetic to ferromagnetic, the magnetization data at 2 K was presented in the form of the Arrott plot, i.e., M^2^ vs. H/M [43], which is shown in Figure 9d. According to the Banerjee criterion [43,44], a magnetic phase transition is expected to have the first-order when the slope of the Arrott plot at M^2^ → 0 is negative, whereas it is of the second-order when the slope is positive at M^2^ → 0. Figure 9d shows that EuTiO_3_ and Eu_0.7_Ba_0.3_TiO_3_ have a negative slope and in contrast, the Co/Ni co-doped samples have a positive slope. The former corresponds to antiferromagnetic transition of the first-order, while the latter is consistent with the ferromagnetic transition that is well-known to be of the second-order.

The cause that turned the Co/Ni co-doped samples from antiferromagnetic to ferromagnetic is not clear. As mentioned in the introduction, doping at the Ti^4+^ site with lower-valence cations usually resulted in such a transition [19,20,21,22,23,25], which was correlated to the oxygen vacancies or the mixed valences of Eu^2+^/Eu^3+^ in some previous works [19,25,45]. However, as shown in Section 3.1, although oxygen vacancies were indeed present in EuTiO_3_ and Eu_0.7_Ba_0.3_TiO_3_, the Co/Ni co-doped samples did not seem to contain oxygen vacancies, because other charge compensation mechanisms than the oxygen vacancies—i.e., the increase in Eu^3+^ numbers with a decrease in Ti^3+^ numbers—were in action for the doping of Co^2+^/Ni^2+^ at the Ti^4+^ site. So, the oxygen vacancies can be excluded as the cause for ferromagnetism in the co-doped samples. In addition, the proposed double-exchange between the mixed valences of Eu^2+^/Eu^3+^ via Ti^4+^ 3d seems not to be the cause, because as shown in Figure 2, the Ni^2+^ co-doped samples had more Eu^2+^/Eu^3+^ pairs than the Co^2+^ co-doped samples, which should lead to a stronger exchange interaction in the former; however, the latter actually had a higher T_C_ (see Table 3). Furthermore, the charge transfer along the Eu^2+^-Ti^4+^-Eu^3+^ route would give rise to a finite DC conductivity, which was not the case, because all the samples in this work had a high resistivity at RT (or lower temperature), as shown in Figure 5.

It is well known that EuTiO_3_ is a G-type antiferromagnet, in which a given Eu^2+^ ion has 6 nearest-neighbor (NN) Eu^2+^ antiparallel and 12 next-nearest-neighbor (NNN) Eu^2+^ parallel [11]. The exchange constant (J_1_) of NN interactions is determined by the competition between the antiferromagnetic superexchange via Ti^4+^ 3d states and an indirect ferromagnetic exchange via Eu^2+^ 5d states, leading to a delicate balance between the antiferromagnetic and ferromagnetic phases [46]. Density functional calculations suggest that pristine EuTiO_3_ has a negative J_1_ and an increase in its lattice parameter leads to a reduced magnitude of J_1_ or even a positive J_1_ at a large enough lattice parameter [46]. Based on the measured values of T_N_ and θ (Table 3), J_1_ and J_2_ (NNN exchange constant) of the samples were estimated by the molecular field theory, which had the values of J_1_/*k* = −0.014 K and J_2_/*k* = 0.035 K (*k*: Boltzmann constant) for undoped EuTiO_3_, in agreement with the previous reports [47]. A greatly reduced J_1_ (i.e., J_1_/*k* = −0.004 K and J_2_/*k* = 0.022 K) was found for Eu_0.7_Ba_0.3_TiO_3_, which might be attributed to its larger lattice parameter according to the above-mentioned calculations [46]. However, the switch from the antiferromagnetic to ferromagnetic interaction in the Co^2+^/Ni^2+^ co-doped samples was not due to a further increase in the lattice parameter, because the structural characterization in Section 3.1 showed that they had a smaller lattice parameter than Eu_0.7_Ba_0.3_TiO_3_. A plausible explanation for such a transition may be the large asymmetric distortion of the Eu–O polyhedron introduced by the aliovalent doping of Co^2+^/Ni^2+^ at the Ti^4+^ site, which promotes the admixture of Eu 5d and 4f states. The odd-symmetry crystal-field term is well-known in optical spectroscopy to cause such an admixture for the rare-earth ions in solids [48]. As a result of the enhanced mixing of the Eu 5d and 4f states, ferromagnetic exchange via Eu^2+^ 5d states prevails over the antiferromagnetic superexchange via Ti^4+^ 3d states, leading to the observed ferromagnetism in the Co^2+^/Ni^2+^ co-doped samples.

## 4. Conclusions

Eu_1−*x*_Ba*_x_*Ti_1−*y*_M*_y_*O_3_ (*x* = 0–0.3, *y* = 0–0.1, M = Co or Ni) samples were synthesized by solid-state reaction at 1400 °C under the flow of Ar+3% H_2_. XRD confirmed that all the obtained samples had a pure phase. XPS analyses showed the mixed valences of Eu^2+^/Eu^3+^ and Ti^4+^/Ti^3+^ in EuTiO_3_ and Eu_0.7_Ba_0.3_TiO_3_, as well as some oxygen vacancies required to keep the charge neutrality. The co-doping of Co^2+^/Ni^2+^ in Eu_0.7_Ba_0.3_TiO_3_ resulted in a reduction in Ti^3+^ numbers but an increase in Eu^3+^ numbers. Such variations in the Eu^2+^/Eu^3+^ and Ti^4+^/Ti^3+^ ratios indicated that oxygen interstitials, rather than vacancies, might be present in the co-doped samples. On the other hand, Ba^2+^ doping led to an increase in lattice parameter due to its larger ionic size than Eu^2+^, whereas the Co^2+^/Ni^2+^ co-doping resulted in smaller lattice parameters because of the combined effects of ionic size and variation in the oxygen-vacancy numbers. Substitution of 30 at% Eu^2+^ by Ba^2+^ led to the appearance of ferroelectricity in Eu_0.7_Ba_0.3_TiO_3_, which persisted in the Co^2+^/Ni^2+^ co-doped samples until the doping level of *y* = 0.05 and 0.10, respectively. Eu_0.7_Ba_0.3_TiO_3_ remained antiferromagnetic with a reduced transition temperature of 3.1 K. The co-doping of Co^2+^/Ni^2+^ turned the samples from antiferromagnetic to ferromagnetic with a transition temperature of 2.98 K and 2.72 K, respectively. The transition could not be explained by the causes proposed in the previous works, i.e., larger lattice volume, oxygen vacancies and mixed valences of Eu^2+^/Eu^3+^. Instead, it was more likely to arise from a large asymmetric distortion of the Eu–O polyhedron introduced by the aliovalent doping, which promotes the admixture of Eu 5d and 4f states.

## Figures and Tables

**Table 1:** Refined lattice parameters of Eu_1−*x*_Ba*_x_*Ti_1−y_M_y_O_3_ (M = Co/Ni).

Sample		a (Å)	R_wp_ (%)	R_p_ (%)	GOF
EuTiO_3_		3.8976(3)	3.82	3.00	1.19
Eu_0.7_Ba_0.3_TiO_3_		3.9340(2)	5.00	3.95	1.21
Eu_0.7_Ba_0.3_Ti_1−*x*_Co*_x_*O_3_	*x* = 0.05	3.93105(3)	4.89	5.15	1.93
	*x* = 0.1	3.93325(18)	5.21	4.06	1.54
Eu_0.7_Ba_0.3_Ti_1−*x*_Ni*_x_*O_3_	*x* = 0.05	3.92930(6)	5.51	4.26	1.11
	*x* = 0.1	3.93075(4)	5.28	4.16	1.27

**Table 2:** P_r_ and E_c_ of Eu_1−*x*_Ba*_x_*Ti_1−y_M_y_O_3_ (M = Co/Ni).

Sample		P_r_ (μC/cm^2^)	E_C_ (kV/cm)
Eu_1−*x*_Ba*_x_*TiO_3_	*x* = 0	0.111	4.77
	*x* = 0.1	0.133	2.25
	*x* = 0.2	0.158	1.53
	*x* = 0.25	1.51	1.19
	*x* = 0.3	2.01	1.21
Eu_0.7_Ba_0.3_Ti_1−*y*_Co*_y_*O_3_	*y* = 0.05	0.408	2.85
	*y* = 0.1	0.0528	1.94
Eu_0.7_Ba_0.3_Ti_1−*y*_Ni*_y_*O_3_	*y* = 0.05	1.07	2.27
	*y* = 0.1	0.415	0.957

**Table 3:** χ_0_, θ, T_C_ and T_N_ of Eu_1−*x*_Ba*_x_*Ti_1−y_M_y_O_3_ (M = Co/Ni).

Sample	χ_0_	θ (K)	T_C_ (K)	T_N_ (K)
EuTiO_3_	0	3.50	--	5.20
Eu_0.7_Ba_0.3_TiO_3_	0	2.55	--	3.05
Eu_0.7_Ba_0.3_Ti_0.95_Co_0.05_O_3_	8.52 × 10^−3^	3.03	2.98	--
Eu_0.7_Ba_0.3_Ti_0.90_Ni_0.10_O_3_	4.62 × 10^−2^	2.48	2.72	--

## Data Availability

Not applicable.
